# Effect of Intranasal Oxytocin Administration on Human-Directed Social Behaviors in Shelter and Pet Dogs

**DOI:** 10.3389/fpsyg.2018.02227

**Published:** 2018-11-16

**Authors:** Gabriela Barrera, Victoria Dzik, Camila Cavalli, Mariana Bentosela

**Affiliations:** ^1^Grupo de Investigación del Comportamiento en Cánidos, Instituto de Ciencias Veterinarias del Litoral, Consejo Nacional de Investigaciones Cientficas y Técnicas (CONICET), Universidad Nacional del Litoral, Santa Fe, Argentina; ^2^Universidad de Buenos Aires, Facultad de Medicina, Instituto de investigaciones Mdicas A Lanari, Buenos Aires, Argentina; ^3^Consejo Nacional de Investigaciones Cientficas y Técnicas, Instituto de investigaciones Médicas, Grupo de Investigación del Comportamiento en Cánidos, Universidad de Buenos Aires, Buenos Aires, Argentina

**Keywords:** oxytocin, gaze, sociability, pet dogs, shelter dogs

## Abstract

A wide variety of evidence has demonstrated that oxytocin is involved in socio-cognitive skills in domestic dogs (*Canis familiaris*). The purpose was to evaluate the effect of oxytocin administration on socio-cognitive abilities in two populations of dogs with different levels of daily human contact: shelter and pet dogs. Additionally, the effect of different doses of oxytocin (i.e., 16 and 24 IU) was assessed. To this end, dogs were tested on two tasks: a sociability test to assess their social responses and a communicative task focused on the learning of gazing responses. Results showed that pet dogs performed better than shelter dogs on the sociability and the gazing tests showing the relevance of dogs’ previous experience and learning when interacting with people. The administration of 16 IU as well as 24 IU of oxytocin improved the performance on the communicative learning task, producing an increment in gaze duration during extinction. This difference was observed in both pet and shelter dogs. Therefore, oxytocin seems to participate in the persistence of this communicative response. However, the treatment did not modify the behaviors during the sociability test. Furthermore, oxytocin appears to be beneficial to increase the communicative abilities of shelter dogs.

## Introduction

An increased interest in the comparison of human-dog interactions on physiological levels has been seen in recent years. Accordingly, investigation of the effects of oxytocin (OT) on companion dogs’ socio-cognitive skills and the related increase of affiliative behaviors has become more relevant.

Oxytocin is a neuropeptide and hormone synthesized in the supraoptic and paraventricular nuclei in the hypothalamus and it is related to a wide range of affiliative and socio-cognitive behaviors in a variety of species (Lee et al., 2009). For instance, OT is involved in cooperative behavior in suricates (*Suricata suricatta*) (Madden and Clutton-Brock, 2010), social behaviors in newborn monkeys (Simpson et al., 2014), social grooming in bats (*Desmodus rotundus*) and chimpanzees (Crockford et al., 2013; Carter and Wilkinson, 2015) and a longer gaze duration toward the eye region among macaques (Dal Monte et al., 2014) and humans (Guastella et al., 2008).

With regard to the human-dog bond, different genetic markers of OT have been associated with human-directed social behaviors in dogs such as microsatellites markers close to the OT receptor gene (OXTR) (Oliva et al., 2016) and OXTR polymorphisms (Kis et al., 2014a; Kubinyi et al., 2017; Oláh et al., 2017; Persson et al., 2017; Turcsán et al., 2017; Konno et al., 2018; Kovács et al., 2018). Furthermore, in relation to epigenetic mechanisms, similar social behaviors were associated with DNA methylations in the promoter region of OXTR in dogs (Cimarelli et al., 2017).

Positive interactions have been shown to increase endogenous OT levels in both species. For example, an increase in OT was observed in both owners and their dogs after approximately 30 min of interaction which included gently stroking and scratching, playing and talking in a positive tone (Odendaal and Meintjes, 2003; Miller et al., 2009). Similar but shorter interactions (i.e., 3–4 min) also increased OT levels in male Labrador dogs (Handlin et al., 2011) and female Beagle dogs (Rehn et al., 2014). Rehn et al. (2014) found that the combination of physical and verbal contact elevated OT levels more than verbal contact only. Thus, both physical and verbal interactions appear to be important for the release of OT during human-dog exchanges. In addition, assistance dogs that experienced a wide variety of interactions with people had higher endogenous OT levels compared to pet dogs (MacLean et al., 2017). Moreover, Nagasawa et al. (2009, 2015) found that the OT levels of both the dogs and their owners, were increased when the dogs gazed more at the owner compared to those that gazed for a shorter time. The authors concluded that the dog’s gazing would likely induce an activation of the OT neuroendocrine system in both of them.

Similar results were found with the administration of exogenous OT in dogs. For example, the application of intranasal OT promoted affiliative behaviors in dogs toward humans as well as toward conspecifics, compared to controls receiving saline (Romero et al., 2014). It also encouraged dogs’ social play with conspecifics (Romero et al., 2015). Additionally, OT improved the performance on a communicative task where dogs had to follow pointing cues to find hidden food (Oliva et al., 2015; Macchitella et al., 2017). In line with this, Nagasawa et al. (2015) demonstrated that the intranasal administration of OT produced an increase in dogs’ gaze duration toward their owners after mutual interaction, although this increment was only seen in female dogs. Furthermore, recent studies found differential breed effects, such as Border Collies looking more toward a human than Siberian Huskies after OT administration, in an unreachable food situation (Kovács et al., 2016b). Moreover, OT administration increased gazing toward people in ancient Japanese dog breeds, which have lower baseline gazing levels than European breeds (Nagasawa et al., 2017). Other changes observed include cognitive bias effects, such as an increase in a positive bias to assess ambivalent stimuli (Kis et al., 2015), looking at projected images on a screen (Kovács et al., 2016a), and gazing at their owners for a longer period when approached in a threatening manner (Hernádi et al., 2015).

The first purpose of our study is to evaluate the effect of the intranasal administration of OT (16 IU) on dogs’ social behaviors during the interaction with a stranger in a sociability test. Secondly, considering that gazing appears to be important for the release of OT during human-dog interactions, we aim to assess the effect of OT on learning a communicative task consisting of gazing at the human face to ask for food. The learning process comprises three phases – an initial phase where the gaze behavior is reinforced with food; an extinction phase where no food is given even if the dog performs the gazing response; and finally, a re-acquisition phase where dogs receive the reinforcement again. We hypothesize that an OT increase will favor the development of social and communicative behaviors in dogs toward humans, given the role of this hormone on social relationships. Furthermore, we propose that these effects could be different according to the dogs’ previous levels of social contact with humans. To this end, in Study 1 we compare the performance of pet and shelter dogs on these tasks. Previous studies have pointed out differences between these populations both in sociability and gazing tasks (Barrera et al., 2010, 2011) and in other communicative tasks (Udell et al., 2008). The poorer performance of shelter dogs on social tasks suggests that such skills could be affected by reward history during ontogeny. As far as we know, there are no studies exploring the effect of OT on shelter dogs, despite the relevance this could have on rehabilitating behavioral problems (Romero et al., 2015; Thielke and Udell, 2015). These results would expand on the knowledge regarding the importance of OT in the human-dog bond, as well as in how it might shape learning and how previous experiences may modulate its effects.

Furthermore, on study 2, we carry out the same tasks in pet dogs after administering a higher dose of OT (24 IU). In previous studies there is no consensus on the OT dose that should be applied, as it ranges from 12 to 40 IU. Therefore, in the current study we set out to investigate whether a higher OT dose for the same tasks would produce an increase on the observed effects. Researching the effects of different OT doses is important given that intranasal OT has been proposed in the applied area as treatment of dog behavioral problems and to improve dog training.

## Ethical Approval

These studies complied with the current Argentine law of animal protection (Law 14.346) and were developed with the approval of the CICUAL (Institutional Commission for the Care and Use of Laboratory Animals) from the Medical Research Institute IDIM UBA-CONICET (Res. Nro. 084-18). All owners and shelter staff signed an informed written consent for the participation of their dogs.

## Study 1

### Materials and Methods

#### Subjects

We assessed 45 dogs, 21 pet dogs (PD), 11 females and 10 males, and 24 shelter dogs (SHD), 10 females and 14 males. Six additional dogs (1 PD and 5 SHD) were excluded from the sample as they showed fear responses to the situation and/or did not reach the criterion for the communicative learning task described below. All of them were adults (2–10 years old) and mongrels with no clear resemblance to any particular breed. Shelter and pet dogs were randomly divided in two groups according to the treatment: OT or Placebo. Therefore, there were 4 groups: pet dogs with OT (PO, *N* = 10), pet dogs with placebo (PP, *N* = 11), shelter dogs with OT (SHO, *N* = 12) and shelter dogs with placebo (SHP, *N* = 12).

All shelter dogs had lived in the shelter for at least 2 years before the testing and their background history was not available. Seventeen SHDs came from a shelter sponsored by the “Asociación Dignidad Animal” (Association of Animal Dignity) in Santa Fe, Argentina; while the remaining seven SHDs were from another shelter belonging to the “Sociedad Protectora de Animales de Santa Fe” (Society for the Protection of Animals of Santa Fe), Argentina. Both shelters offered similar accommodations for the dogs, with large open separate areas (10 m long × 8–10 m wide), holding between 5 and 15 dogs per sector. Their contact with shelter staff was limited to feeding and cleaning activities. They were all in good health and none of them had previously participated in a study.

The selection criteria used for PD was to choose dogs that had spent most of their lives in a household and had daily interaction with their owners inside the house. A total of 4 pet dogs had already been assessed in other tasks (self/inhibitory control).

Dogs had free access to water and the last meal before the communicative learning task had been received between 4 and 6 h earlier.

#### Administration

The dogs received intranasal OT (16 IU) Syntocinon-Spray (Novartis) or saline solution as placebo, 40 min before the sociability test and the communicative learning task. This time frame was selected as it is the time it takes for OT to reach the brain (MacDonald et al., 2011; Quintana et al., 2015).

Immediately after application, the animals remained in their homes or enclosures for 30 min while performing their usual activities and not interacting with the humans. They were then taken to the evaluation room for 10 min so that they became familiar with the surroundings, accompanied by an unfamiliar experimenter that was indifferent to the dog. After the 10 min of habituation, this experimenter left the room and the testing began immediately after.

#### Procedure

The procedure comprised one test (sociability test) and one task (communicative learning task). They were always carried out in this order and on the same day. The interval between them was 2 min. The fixed order of procedures was to ensure the assessment of the animals’ initial reaction to the presence of a stranger in the sociability test. In addition, the sociability test acted as a familiarization to the Experimenter (E). Both tests were performed by the same female E who was unknown to the dog and blind to the dogs’ treatment (OT-placebo). The tests were scheduled in a quiet familiar room in the dogs’ usual environment (home or shelter).

##### Sociability test

###### Apparatus

The test was carried out in a closed room where there was a chair placed against a wall. Tape marks on the floor 1 m away from the chair were used to later determine the distance kept by the dogs. Only the E and the dog were present during testing. The evaluation was videotaped by a camera (Sony DCR 199 SX-85) located on a tripod.

###### Procedure

The sociability test was the same as Jakovcevic et al. (2012). It was divided into two 2 min phases: (a) Passive phase: The E entered the room and sat on the chair reading a book. If the dog made physical contact with her, E petted its head, neck, or back twice and then withdrew her hand. During this phase the E did not make visual contact with the dog. (b) Active phase: The E stood up, called the dog by its name and made visual contact with it. If the dog approached, E interacted by petting and talking to it. If the dog did not approach, E called it three times. If the dog approached and then went away, E called it up to three times. During this second phase, E stayed in the same place to avoid possible fear reactions in the dog.

The following variables were registered for both phases (passive and active):

Time (s) the subject remained near the E (<1 m distance).

Time spent in physical contact (s) between the E and the subject.

##### Communicative learning task

The task was the same as in Barrera et al. (2010) and Jakovcevic et al. (2010).

###### Apparatus

A container with the reward was placed on a high shelf, so that it was visible to the dogs but out of their reach. The E stood beside the container. All trials were video-taped by a Sony DCR 199 SX-85 camera. The person taping the trial ignored the dog and was located behind the E, so as to be able to film the dog’s gaze and head position. The task was performed in the same area as the sociability test. Each session involved the dog, the E, and the assistant operating the camera. The reward were pieces of cooked liver.

###### Procedure

The procedure consisted of three phases:

###### Acquisition (ACQ)

The phase began after a warm up in which the E called the dog by its name, actively sought physical contact and gave it three pieces of liver. Acquisition trials started with E standing beside the food container and once again calling the dog by its name and giving it a piece of liver. Gazing at E’s face for at least 1s was reinforced at every occurrence. Usually, dogs moved their gaze from E’s face to her hand as soon as E reached for the food, and a new reinforcer was delivered when the dog turned its gaze back to E’s face for 1 s. A selection criterion where dogs had to respond to their names and gaze at E at least four times during the last acquisition trial was established. Dogs received three 2 min trials of differential reinforcement of gazing at the E. The inter-trial interval (ITI) lasted 2 min.

###### Extinction (EXT)

The interval between acquisition and extinction phases lasted 2 min. Three 2 min extinction trials were performed. The ITI lasted 2 min. During this phase the dogs never received food, so the gazing response was no longer reinforced. The E and the food remained in the same place as in previous trials.

###### Reacquisition (RA)

Two minutes after extinction, the dogs received one trial of reacquisition which was identical to the acquisition trials. This phase discarded potential satiety or fatigue effects.

During acquisition, extinction and reacquisition trials, the E remained in the same position gazing at the dog’s face.

The dependent variable in all trials was the cumulative gaze duration (s) toward the E‘s face. To measure this, a stopwatch was activated each time the dogs directed its head/gaze to the E’s face and stopped when the direction of the head/gaze changed/they looked away.

### Data Analyses

For both tasks, an experimenter blind to the dogs’ treatment (OT-placebo) measured 100% of the video-taped material. Additionally, in order to assess interobserver reliability, a second observer analyzed 40% of the material. We calculated Pearson’s coefficients of correlation for all the measures and they showed high reliability for both tasks (Sociability test: *r* > 0.99, *p* < 0.0001, *n* = 18; Communicative learning task: *r* > 0.92, *p* < 0.0001, *n* = 18).

The sociability test was analyzed using generalized linear mixed modeling on the time spent near and in contact. The distribution of the variables (seconds) was specified as normal and related to the fixed factors through the identity link function. Phase (passive and active), treatment (OT, placebo), housing (SHD, PD) and sex (male, female) were included as fixed factors into the model. Additionally, six two-way interactions were specified: three interactions resulting from crossing the within-subject factor (i.e., phase) with each of the between-subjects factors (i.e., treatment, housing and sex), and three interactions resulting from pair-crossing the between-subjects factors. The random effect structure for near and contact was by-participants intercepts.

For gazing, analyses, statistical software and fixed and random factors’ specifications were the same as in the sociability test. It is important to note, however, that the factor phase was composed of three levels (acquisition, extinction, and reacquisition) in this case, rather than two, as in the previous task. The random effects’ structure included intercepts to account for variability across participants and across trials, since data collection took place in seven different trials.

All tests were two-tailed, α = 0.05 and were carried out in SPSS 22.0.

### Results

#### Sociability Test

The mean duration (s) and standard deviations of time spent near and in contact are reported in Table 1. The analyses yielded a similar pattern for both variables: a significant main effect of housing: near *F*(1,77) = 5.05, *p* = 0.027; contact *F*(1,77) = 6.99, *p* = 0.01, and a significant main effect of phase: near *F*(1,77) = 50.91, *p* < 0.0001; contact: *F*(1,77) = 65.74, *p* < 0.0001, were found. As shown in Table 1, the average duration of social behaviors in the PD group and the active phase condition were significantly longer than those from the SHD group and the passive phase condition, respectively. The rest of the factors did not yield significant effects, *p* > 0.05.

**Table 1:** Near and contact to the E on sociability test of study 1.

	Passive phase		Active phase	
	Near	Contact	Near	Contact
SHO	**14.48** ± 24.82	**4.55** ± 8.78	**78.16** ± 50.96	**67.76** ± 52.94
PO	**59.36** ± 43.76	**43.01** ± 46.93	**111.45** ± 9.53	**111.36** ± 9.65
SHP	**29.85** ± 37.84	**16.63** ± 31.68	**74.99** ± 47.86	**72.85** ± 46.83
PP	**28.02** ± 36.32	**16.46** ± 36.31	**84.53** ± 54.50	**84.53** ± 54.50

*Both behaviors are calculated in ratios of the cumulative durations in seconds on each phases (passive and active). Cells indicate the mean (in bold) and the standard deviation of the time ratio that SHO (shelter dogs with OT); PO, pet dogs with OT; SHP, shelter dogs with placebo; and PP, pet dogs with placebo; spent near and contact to the E on each phases.*

#### Communicative Learning Task

**FIGURE 1:**
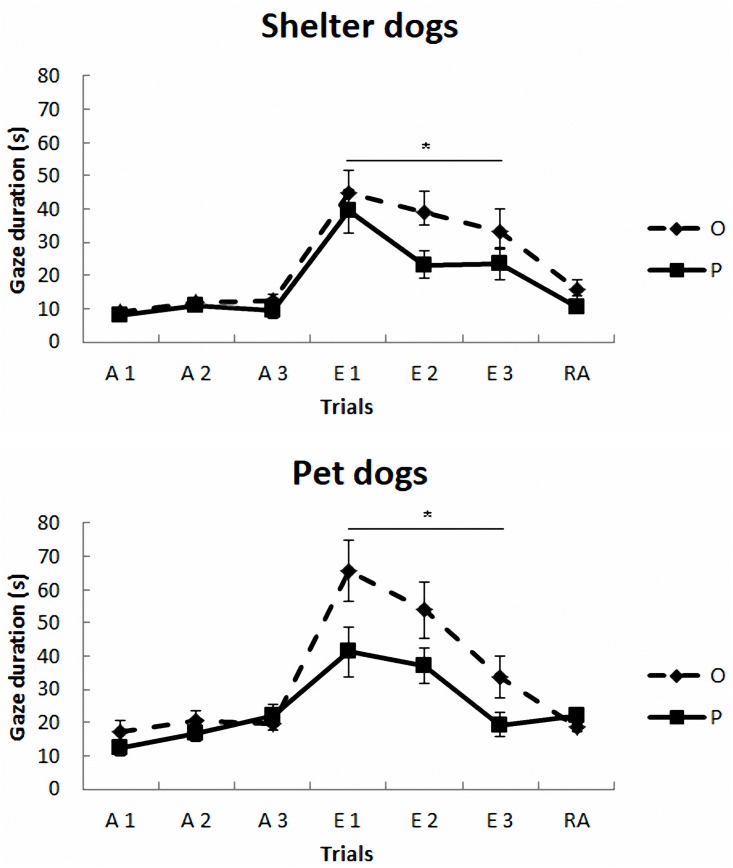
Communicative learning task of the Study 1. Gaze duration (s) in acquisition (ACQ), extinction (EXT), and reacquisition (RA) trials for the groups SHO (shelter dogs with OT), PO (pet dogs with OT), SHP (shelter dogs with placebo) and PP (pet dogs with placebo) (means ± SEM). Dogs were required to gaze at the experimenter for 1 s to receive food in the acquisition and reacquisition phases. During extinction no food was delivered. ^∗^*p* < 0.05.

The mean duration (s) and standard deviations of gazing are reported in Figure 1 and Table 2. The model yielded a significant main effect of housing, *F*(1,297) = 5.78, *p* < 0.017, so that the average gaze duration from the PD group was significantly longer than that from the SHD group. The model also yielded a significant main effect of phase, *F*(2,297) = 8.52, *p* < 0.0001. The *post-hoc* Bonferroni comparisons indicated that the average gaze duration in extinction was significantly longer than in the acquisition phase, *t*(297) = 3.94, *p* < 0.0001, and reacquisition, *t*(297) = 2.53, *p* = 0.002. As expected, gaze durations from phase 1 and phase 3 did not differ significantly, *t*(297) = 0.25, *p* = 0.8. Finally, the model also revealed a significant treatment × phase interaction, *F*(2,297) = 7.65, *p* < 0.001, so that the group that received OT showed longer gaze times than the group that received placebo, but only in phase 2, *t*(297) = 3.63, *p* < 0.0001. Gaze duration did not differ in phase 1 and phase 3 as a function of treatment, *p* > 0.05. The other comparisons were non-significant, *p* > 0.05.

**Table 2:** Mean (in bold) and SD of the gaze duration (s) across trials for each group of Study 1.

Group	Acq 1	Acq 2	Acq 3	Ext 1	Ext 2	Ext 3	Reacq
SHO	**8.92** ± 5.78	**11.72** ± 5.79	**12.67** ± 5.92	**45.03** ± 22.95	**39.24** ± 21.41	**33.10** ± 23.62	**15.68** ± 9.85
SHP	**7.94** ± 6.11	**11.00** ± 6.69	**9.41** ± 7.12	**39.33** ± 22.52	**23.26** ± 14.75	**23.43** ± 15.56	**10.73** ± 6.87
PO	**17.28** ± 10.09	**20.79** ± 9.03	**19.82** ± 6.10	**65.64** ± 29.52	**54.05** ± 26.76	**33.71** ± 20.46	**18.94** ± 5.52
PP	**12.53** ± 8.24	**16.89** ± 8.71	**22.27** ± 10.54	**41.34** ± 24.74	**37.34** ± 17.64	**19.46** ± 12.23	**22.03** ± 7.63


*Acq, acquisition; Ext, extinction; Reacq, reaquisition; SHO, shelter dogs with OT; SHP, shelter dogs with placebo; PO, pet dogs with OT; and PP, pet dogs with placebo.*

These results indicate that PD performed better than SHD on both the sociability and the communicative learning task. This could be related to the dogs’ prior history as well as SHD low daily contact with humans, which limits their possibilities to learn communicative responses.

Secondly, in both PD and SHD dogs, the administration of OT increased the duration of gazing to the human face to ask for food during the extinction phase, when this response was no longer successful. This suggests that OT modulates the persistence of this learned communicative response. Conversely, no OT effects were observed during the acquisition and reacquisition phases. Probably, the delivery of food masks any potential OT effect. Additionally, no differences between these dog populations were observed during the sociability test. One possibility is that the dose used in this study was insufficient to produce differences in this test so in Study 2 we set out to evaluate the effects of a higher dose of OT.

## Study 2

The aim of this study was to replicate the findings of Study 1, contributing to a larger sample size and using a higher OT dose. Given that OT affected both pet and shelter dogs similarly on the previous study, in this case only pet dogs were evaluated.

### Materials and Methods

#### Subjects

We assessed 41 dogs, 22 females and 19 males. Four additional dogs were excluded from the sample as they showed fear responses to the situation and/or did not reach the criterion for the communicative learning task. All of them were adults of 1–10 years of age (mean age: 4.54 years, SD: 2.55 years old) and mongrels with no clear resemblance to any particular breed. The dogs were randomly assigned into two groups: OT or Placebo. Therefore, there were 2 groups: pet dogs with OT (O, *N* = 20) and pet dogs with placebo (P, *N* = 21).

#### Procedure

The procedure was exactly the same as in Study 1, except that the OT administration was of 24 IU.

#### Data Analyses

Regarding inter-observer reliability, the procedure was the same than in Study 1, Pearson’s coefficients of correlation for all the measures were run and they showed high reliability for both tasks (Sociability test: *r* > 0.98, *p* < 0.0001, *n* = 17; Communicative learning task: *r* > 0.93, *p* < 0.0001, *n* = 17).

Data were examined using generalized linear mixed models. For the sociability test, two identical generalized linear mixed models were specified, one for each outcome variable (near and contact). The models included phase (passive, active), treatment (OT, placebo), and sex (male, female) as fixed factors. The models also included all possible interactions (i.e., three two-way interactions and a three-way interaction) as additional fixed factors and by-participants random intercepts (α = 0.05).

For the communicative learning task, gazing was entered as the outcome variable. Phase (acquisition, extinction and reacquisition), treatment (OT, placebo), and sex (male, female) were entered as fixed factors into the model. The fixed effects’ structure also included three two-way interactions, resulting from pair-crossing the three fixed factors, and a three-way interaction including all factors (α = 0.05). The random effects’ structure included intercepts to account for variability across participants and across trials, since data collection took place in seven different trials. The Satterthwhaite approximation method was used to estimate the degrees of freedom due to different cluster sizes in the between and within-subjects factors. When necessary, additional analyses were conducted using *post-hoc* paired comparisons (Adjusted Sequential Bonferroni).

### Result and Discussion

#### Sociability Test

In the sociability test (see Table 3), the analyses yielded significant effects of phase on both outcomes (near *F*(1,74) = 30.06, *p* < .0001; contact *F*(1,74) = 75.96, *p* < 0.0001). As shown in Table 2, the average duration of the behaviors in the active phase was significantly longer than those from the passive phase. The rest of the fixed factors did not yield significant effects, *p* > 0.05.

**Table 3:** Near and contact to the E on sociability test of study 2.

	Passive phase		Active phase	
	Near	Contact	Near	Contact
O	**41.76** ± 43.82	**19.98** ± 29.10	**87.09** ± 44.76	**86.20** ± 44.82
P	**54.78** ± 48.33	**26.59** ± 36.54	**83.84** ± 48.75	**83.72** ± 48.97

*Cumulative durations in seconds of each phase (passive and active). Cells indicate the mean (in bold) and the standard deviation of the time that O (oxytocin) and P (placebo) spent near and in contact to the E during each phase.*

#### Communicative Learning Task

**FIGURE 2:**
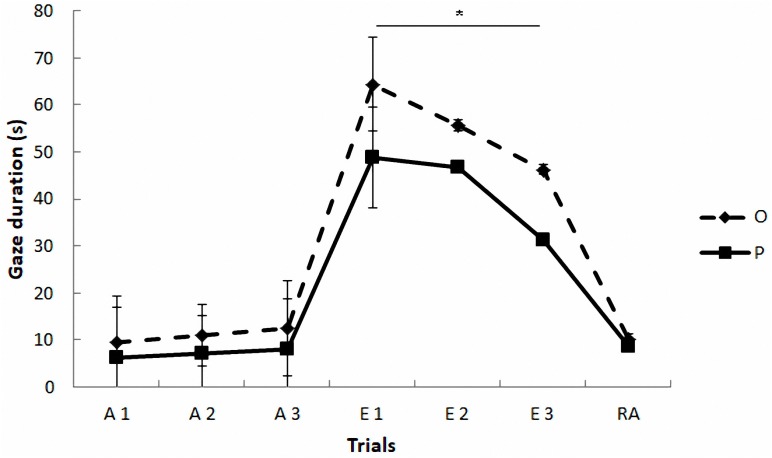
Communicative learning task of the Study 2. Gaze duration (s) in acquisition (ACQ), extinction (EXT), and reacquisition (RA) trials for the groups O (OT) and P (placebo) (means ± SEM). Dogs were required to gaze at the experimenter for 1 s to receive food in the acquisition and reacquisition phases. During extinction no food was delivered. ^∗^*p* < 0.05.

Regarding the gazing task (see Figure 2 and Table 4), the mixed model revealed a significant effect of phase on gazing, *F*(2,4) = 31.37, *p* = 0.004. The paired comparisons indicated that gaze duration was longer in extinction phase than in acquisition (*p* = 0.005) and reacquisition phases (*p* = 0.013), which did not differ between them (*p* = 0.97). The model also yielded a marginally significant effect of treatment, *F*(1,42) = 3.79, *p* = 0.058, and a significant treatment by phase interaction, *F*(2,234) = 6.21, *p* = 0.002, so that the OT treatment led to longer gaze times than the placebo treatment, but only in extinction (Sequential Bonferroni, *p* < 0.0001). No significant effects were found as a function of the sex of the participants *F*(1,1016) = 3.14, *p* = 0.08, and the rest of the interactions (*p* > 0.05).

**Table 4:** Mean (in bold) and SD of the gaze duration (s) across trials for each group of Study 2.

Group	Acq 1	Acq 2	Acq 3	Ext 1	Ext 2	Ext 3	Reacq
**O**	**9.62** ± 5.31	**10.94** ± 4.92	**12.52** ± 4.59	**64.37** ± 20.09	**55.56** ± 27.60	**46.24** ± 27.67	**10.16** ± 4.31
**P**	**6.38** ± 5.14	**7.09** ± 5.89	**8.17** ± 4.74	**48.87** ± 22.38	**46.59** ± 17.96	**31.38** ± 19.32	**8.55** ± 3.75

*Acq, acquisition; Ext, extinction; Reacq, reaquisition; O, oxytocin; P, placebo.*

The results of Study 2 are consistent with those of Study 1. The administration of OT had no effects on the sociability test. However, it increased the duration of gazing toward the human face during extinction. Therefore, a higher dose of OT did not increase the previously observed effects during these tasks.

## General Discussion

The results of our studies demonstrate that pet dogs exhibit more approach and physical contact behaviors toward a stranger than shelter dogs. This is in disagreement with our previous study in which shelter dogs spent more time near a stranger than pet dogs (Barrera et al., 2010). Conversely, Shin and Shin (2017) did not find any difference in the sociability of shelter and companion dogs. It is possible that the characteristics of the shelters were different and this would affect in a different way the behavior of the dogs. It is necessary to evaluate shelter dogs with several standardized tests in order to clarify their relative levels of sociability compared to pet dogs. Moreover, the responses were higher during the active phase compared to the passive one. This is probably related to the active E’s attitude during the second phase that promotes the appearance of more social behaviors in dogs.

Furthermore, we found that the intranasal administration of OT (16 IU or 24 UI) does not change the reactions to a stranger in the sociability test. These findings are contrary to previous studies in dogs that show that OT modifies affiliative behaviors, particularly approach and physical contact (Mitsui et al., 2011; Romero et al., 2014). This difference may be due to the fact that the standardized experimental situation, especially in the passive phase, failed to promote the appearance of more social responses. Additionally, the presence of a stranger may have triggered less social responses than when the owner is present, even after OT administration. There is evidence that OT may have differential effects in the case of a familiar person compared to a stranger (see De Dreu, 2012; Persson et al., 2017). Finally, the test used here was probably not sensitive enough to detect differences between groups, especially during the active phase in which the experimenter promotes interaction and thus facilitates the appearance of social responses. This could have equated the performance of dogs from both groups.

However, our results are in line with recent studies that did not find OT to improve proximity seeking and contact toward people (Nagasawa et al., 2017; Thielke et al., 2017). It has been suggested that OT could increase social responses according to the context and its perception as positive or threatening (Bartz et al., 2011; Turcsán et al., 2017). As stated by Buttner (2016), the effect of OT is complex and interacts with multiple modulating factors, such as the context, the sex of the dogs and the stress levels.

On the other hand, pet dogs gazed more at the human face to ask for food than shelter dogs. This replicates our previous findings (Barrera et al., 2011) and may be related to the fact that shelter dogs have a deficit in some communicative responses associated with a long learning history with low human contact. These findings are consistent with the Two Stage hypothesis (Udell et al., 2010) that states that dogs’ communicative abilities are not only the product of domestication, but of learning and experiences during ontogeny.

Regarding the effect of treatment, no significant difference was found during acquisition. The presence of food probably made both groups react similarly, by making them reach a ceiling level in their response. Moreover, it could be argued that the experimenter’s hand reaching toward the food became a conditioned reinforced by its repeated pairing with it, signaling the availability of reinforcement shortly. Because of this, dogs rapidly started gazing toward the hand before receiving the food and this may have contributed to the appearance of a ceiling effect on the gazing behavior toward the experimenter’s face.

Conversely, we have demonstrated that OT increases the duration of the gaze at the human face during the extinction phase, when the animals are no longer reinforced to gaze at the person. This effect was observed with the administration of both 16 and 24 IU of OT. Oxytocin probably increases not only the duration of the gaze *per se* but also its persistence when this response is unsuccessful, so that the most significant differences are seen during the extinction phase when dogs receive no food.

Contrary to our predictions, the OT effect was similar on pet and shelter dogs. This suggests that the increase of gazing as a requesting behavior, appears besides the level of daily social contact of the dogs.

The differences observed during extinction, may also be due to the effect of OT on decreasing stress and anxiety (e.g., Kis et al., 2014b). For instance, OT reduces the activity of the hypothalamic-pituitary-adrenal (HPA) axis (Uvnas-Moberg and Petersson, 2005). In addition, increased levels of OT were associated with stressful events (Engert et al., 2016; Li et al., 2016). Given that the extinction phase produces emotions similar to stress (Konorski, 1959), the anxiolytic effect of OT could facilitate the persistence of the gazing response.

It has been suggested that this stress reducing effect of OT is related to a drop in attention and vigilance toward socially threatening stimuli (Kis et al., 2017; Somppi et al., 2017; Turcsán et al., 2017). For instance, after OT treatment, dogs gazed less toward angry faces in comparison to happy or neutral ones (Kis et al., 2017; Somppi et al., 2017). Additionally, human gaze may be interpreted as threatening or positive according to the context (Tops et al., 2018). Taking this into account, one possible explanation for these results could have been that dogs interpreted the human gaze as threatening and the administration of OT helped reduce this effect. However, the context of this study was appetitive as dogs receive food for gazing at the human face. Therefore, OT effects appear to be related to affiliative mechanisms and not to a decrease of negative stimuli during the test.

In relation to this, one important issue to consider is that shelter dogs lived at least 2 years in a potentially stressful environment. Therefore, the interaction between OT and stress could influence the performance of dogs from this group. Further research involving physiological assessments of the basal levels of stress (i.e., heart rate, cortisol levels) in shelter and pet dogs is needed to confirm this hypothesis.

Finally, it should be noted that the sex of dogs did not affect any of the responses assessed, although in some previous studies OT effects were only noted in female dogs (Nagasawa et al., 2015; Oliva et al., 2015).

One limitation of this study is the little information available about the shelter dogs’ history. For instance, it is impossible to know whether they ever lived in a family, if they had past traumatic experiences with people or if they were surrendered because of behavioral problems. Another limitation is that, when generalizing these results, it must be considered that only mongrel dogs were evaluated in these studies. Therefore, it is not possible to conclude whether the observed differences would appear in dogs of particular breeds, especially given that the effects of OT have been shown to be differential across breeds (e.g., Kis et al., 2014a; Kovács et al., 2016b; Nagasawa et al., 2017). Finally, it must be taken into account that in this study only two OT doses were evaluated, while in the literature even higher doses (i.e., 40 IU) are used. It would be interesting to compare the effects of more OT doses in further studies.

In sum, our findings demonstrate that OT is related to the persistence of gazing at the human face when food is inaccessible in pet dogs. We have demonstrated for the first time that shelter dogs also increase their gaze at the human face to ask for food after OT application. This finding is particularly relevant for two reasons. First, prior studies indicated that shelter dogs gaze less than pet dogs. This suggest a communicative response deficit in shelter dogs that may be reversed with the administration of OT. Second, gazing is a key response in the human-dog bond (e.g., Miklósi et al., 2003), so it is critical that shelter dogs acquire this response to encourage the possibility of successful adoption and reintegration to family life. In the future, it would be interesting to assess the effect of OT on other communicative tasks in shelter dogs. Moreover, we found that two different doses of OT produce similar effects on gazing to the human face as a communicative response to request food.

The present results show that it is possible to analyze the neurophysiological mechanisms and, particularly, the role of OT in the human-dog bond, by using a minimally invasive method like nasal administration. This helps to examine the relationship between OT and a wide range of socio-cognitive responses in different dog populations as well as provides valuable information on the potential therapeutic value of OT.

## Author Contributions

GB, VD, CC, and MB designed the studies and participated in the data collection. GB, VD, and CC measured the videos. MB and GB analyzed the data. All authors contributed in the writing of manuscript, collaborated to its revision for important intellectual content, read and approved the submitted version.

## Conflict of Interest Statement

The authors declare that the research was conducted in the absence of any commercial or financial relationships that could be construed as a potential conflict of interest.
